# Mechanical Properties, Microstructure, and Chloride Content of Alkali-Activated Fly Ash Paste Made with Sea Water

**DOI:** 10.3390/ma13061467

**Published:** 2020-03-23

**Authors:** Salman Siddique, Jeong Gook Jang

**Affiliations:** Division of Architecture and Urban Design, Institute of Urban Science, Incheon National University, 119 Academy-ro, Yeonsu-gu, Incheon 22012, Korea; salmansiddique@inu.ac.kr

**Keywords:** sea water, alkali-activated material, fly ash, chloride, microstructure

## Abstract

The aim of the present study is to investigate the potential of sea water as a feasible alternative to produce alkali-activated fly ash material. The alkali-activated fly ash binder was fabricated by employing conventional pure water, tap water, and sea water based alkali activating solution. The characteristics of alkali-activated materials were examined by employing compressive strength, mercury intrusion porosimetry, XRD, FT-IR, and ^29^Si NMR along with ion chromatography for chloride immobilization. The results provided new insights demonstrating that sea water can be effectively used to produce alkali activated fly ash material. The presence of chloride in sea water contributed to increase compressive strength, refine microstructure, and mineralogical characteristics. Furthermore, a higher degree of polymerization on the sea water-based sample was observed by FT-IR and ^29^Si NMR analysis. However, the higher amount of free chloride ion even after immobilization in sea water-based alkali-activated material, should be considered before application in reinforced structural elements.

## 1. Introduction

Fresh water is the most consumed natural resource on the planet. Its diverse use in sustaining life and lifestyle of humans has made it one of the most precious resources. On the other hand, the cement concrete remains the largest consumed man-made resource. Two major environmental concerns being raised on use of cement are its huge carbon and water footprint [1,2]. As a countermeasure against the high carbon footprint of Portland cement, the development of alkali-activated materials (AAM) is considered as a cementless binder solution. However, the higher cost of production of AAM has been a limiting factor in its widespread use. One of the major subsections in higher cost of AAM is usually due to the use of alkali activating solution with highly refined chemicals. Furthermore, the use of pure/distilled water is a major concern due to dwindling fresh water resources. Thus, exploring suitable replacement of pure/distilled water to produce AAM is one of the important aspects of research.

Sea water is one of the largest untapped available sources of water. Its use in manufacturing traditional cement concrete is very limited due to the presence of aggressive compounds and higher chloride based salt compounds [3]. It was reported in various studies that use of supplementary cementitious materials along with sea water as mixing water, lowered the probability of chloride induced corrosion in steel rebar [4,5,6,7]. Furthermore, recent studies have been carried out to observe the influence of sea water on characteristics of AAM fabrications by utilizing ground granulated blast furnace slag. Li et al. [8] produced slag-based alkali-activated concrete containing sea water and studied its mechanical properties after thermal exposure. They reported no obvious influence of sea water on thermal properties [8]. On the other hand, few studies have reported slightly negative influence of sea water on the properties of AAM made with sea water. Shi et al. [9] developed calcium silicate slag-based AAM by utilizing artificial sea water. They reported that no negative influence of sea water was observed on the formation of amorphous calcium aluminosilicate hydrate (C–A–S–H) gel. However, the magnesium ions present in the artificial sea water also form magnesium silicate hydrate (M–S–H) which lowered compressive strength [9]. Furthermore, hindrance in alkali activation process was reported due to the coating effect on particles [9]. Yang et al. [10] developed slag-based AAM produced by artificial sea water. The compressive strength was slightly low for sea water based samples. However, improved durability against chloride ion was observed [10]. It should be noted that in studies by Shi et al. [9] and Yang et al. [10] the sea water used for investigations was prepared in the laboratory as per the guidelines of ASTM D1141 [11]. Nevertheless, as discussed in the previous studies sea water has almost negligible effects on the polymerization products of slag-based AAM [8,9,10]. However, an important aspect of high amount of chloride ions present in the sea water samples was not given due consideration in previous studies.

The objective of this study is to investigate the properties of alkali-activated fly ash materials produced by pure water, tap water, and sea water. The feasibility of sea water as alkali-activating solution and its effect on properties of alkali-activated fly ash material were assessed by a suitable experimental program. The compressive strength, microstructural characteristic and mineralogy of alkali-activated fly ash were investigated to assess the influence of various types of water sources. Specific attention was given to investigate the free chloride content of the alkali-activated fly ash.

## 2. Experiments

### 2.1. Materials and Sample Preparation

**Figure 1:**
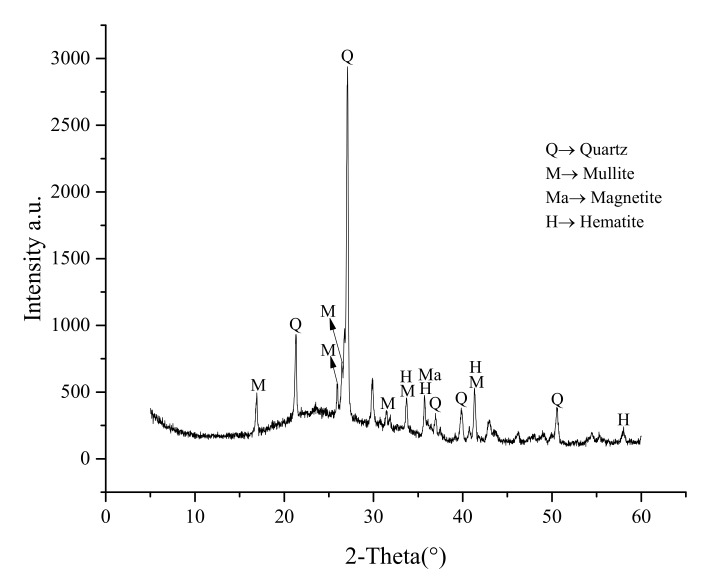
XRD spectra of fly ash.

The class F fly ash (as per ASTM C618 [12]), a byproduct of the Dangjin thermal power plant in South Korea, was used as a precursor for AAM. The chemical composition of fly ash as obtained by X-ray fluorescence (Model: Philips PW2404, Philips, Amsterdam, Netherlands) is presented in Table 1. Figure 1 presents the X-ray diffraction spectra of fly ash.

The presence of quartz, mullite, magnetite, and hematite was observed. 9M NaOH solution and Na_2_SiO_3_ (SiO_2_ = 29 wt.%, Na_2_O = 10 wt.%, and H_2_O = 61 wt.%) were mixed to produce alkali activating solution, and the mass ratio of 9M NaOH and Na_2_SiO_3_ was maintained at 1:1 throughout the study. The silica modulus of alkali activating solution was 2.9. For making 9M NaOH solution, three different types of water solvents were used namely, pure water (also known as distilled water), tap water and sea water. The sea water was collected from the West Sea of Korea and the composition of all three types of water is shown in Table 2.

Ion chromatography was employed to observe the composition of all three types of water. It can be observed that chloride has a major presence in the sea water sample. In addition, the cation concentration of all three water types was obtained by employing inductively coupled plasma atomic emission spectroscopy (ICP-AES; Model: Jobin Yvon Ultima 2, Horiba, Kyoto, Japan) and is shown in Table 3.

The total organic carbon of all three water measured by employing total organic carbon analyzer (Model: vario TOC cube, by ELEMENT AR, Langenselbold, Germany) was found to be 1.08 ppm for PW, 2.82 ppm for TW, and 2.22 ppm for SW. The 24 h cool-off period was considered for the alkali-activating solution before casting.

A constant activator to fly ash ratio of 0.5 was maintained throughout the study to fabricate the AAM. To produce 1 L volume of paste, 1197 g of fly ash and 598 g of alkali-activating solution were mixed. The similar table flow value of 200–210 mm was observed for all the alkali-activated paste mixes. The samples were cast into mold of 50 mm cubic side. The samples were then sealed in plastic cover to prevent evaporation and heat cured for 24 h at 60 °C in hot air oven. After the required heat curing the samples were kept in the open laboratory environment until the specific testing dates. The nomenclature followed in the study denotes PW—for pure water based samples, TW—for tap water based samples, and SW—for sea water based samples.

### 2.2. Testing and Characterization Protocols

The compressive strength test of hardened AAM was carried out at 3, 7, 14, 21, and 28 days on a 3000 kN universal testing machine (DUT-D100, Daehan, Korea) at a loading rate of 1000 N/s as per the guidelines of ASTM C109 [13]. The average of three test results is reported along with the standard deviation. The microstructural characteristics of AAM were studied by employing micromeritics mercury intrusion porosimetry (MIP; Model: micromeritics, Micromeritics Instrument Corporation, Norcross, GA, USA). The pressure range from 30 to 60,000 psi was selected for pore size detection. The mercury surface tension was kept at 485 dynes/cm. The crystalline phases present in samples were identified by carrying out X-ray diffractometry (XRD). XRD was conducted on a PanAlytical device (Malvern Panalytical Ltd, Malvern, UK) with a scan range of 5° to 60° (scan speed of 0.2°/min). The Fourier transform infrared (FT-IR) spectra were collected from 400 to 4000 cm^−1^ at a resolution of 4 cm^−1^. The FT-IR spectra of AAM were obtained by using Vertex 80v (Bruker, Billerica, MA, USA). The ^29^Si MAS-NMR (Bruker, Billerica, MA, USA) analysis was conducted under the conditions of 79.51 MHz at a spinning speed of 11.0 kHz, a pulse length of 30° (1.6 μs) and a relaxation delay of 20 s for the quantitative study. An external sample of trimethylsilyl silane at –135.5 ppm with respect to trimethylaluminium (TMA) at 0 ppm was used to refer chemical shifts. The amount of free chloride ion was measured by ion chromatography carried out on ICS-1600 (Model: Dionex, Sunnyvale, CA, USA). All samples at the age of 28 days were treated with acetone and were then dried by vacuum desiccator. The samples for XRD, FT-IR, NMR, and ion chromatography were ground to pass 150 µm, whereas bulk samples were used for MIP measurements. For ion chromatography measurements, 20 g of ground paste sample was mixed with 100 mL deionized water and the filtered leachate was then examined for quantification of chloride content.

## 3. Results and Discussion

### 3.1. Compressive Strength

**Figure 2:**
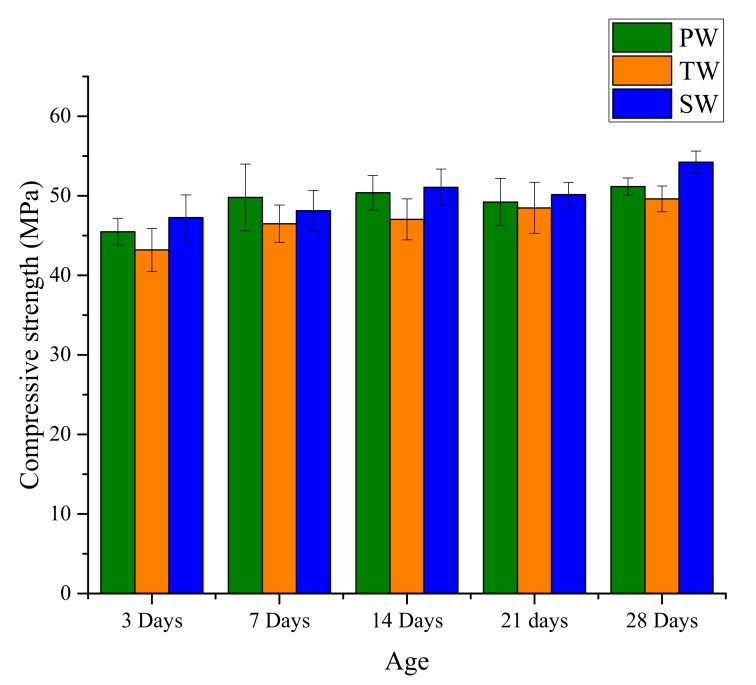
Compressive strength development of alkali-activated materials (AAM) produced with different types of water.

Figure 2 shows the compressive strength of AAM samples measured at different testing age. It can be observed that at the age of 28 days the AAM samples with sea water displayed the highest compressive strength.

However, AAM samples made with all three different water types have quite similar compressive strength at 28 days, i.e., PW-51.16 MPa, TW-49.62 MPa, and SW-54.22 MPa. It should be noted that in case of PW the strength development peaks at 7 days of curing with just marginal increase (2.7%) at 28 days. However, in the case of TW and specifically SW an increase of 6.7% and 12.7%, respectively, was observed between the testing age of 7 and 28 days. Similar results of higher compressive strength for sea water based alkali-activated slag were reported in studies by Rashad and Ezzat [14] and Yang et al. [10]. On the other hand, Shi et al. [9] reported that the use of sea water resulted in lower compressive strength of calcium silicate slag-based AAM.

In the case of SW mixes, the higher amount of sulfate and sodium ions can accelerate the polymerization process and ensure closure of pores [14]. It should be noted that in the present study the amount of calcium in fly ash is only 5%, thus the formation of CaCl_2_ might have been restricted. Nevertheless, the limited formation of CaCl_2_ can also accelerate an early age polymerization [10]. Furthermore, SW registered a higher pH value than TW and PW (see Table 2). The polymerization of AAM could be influenced by the pH value and a higher pH could promote the polymerization process. In addition, a higher pH value can reduce the probability of corrosion by the formation of passive layer around steel rebar. Another possible aspect of SW effect is the higher amount of total dissolved solids which can improve the compressive strength by reducing the porosity between the crystalline polymers.

### 3.2. Porosity

**Figure 3:**
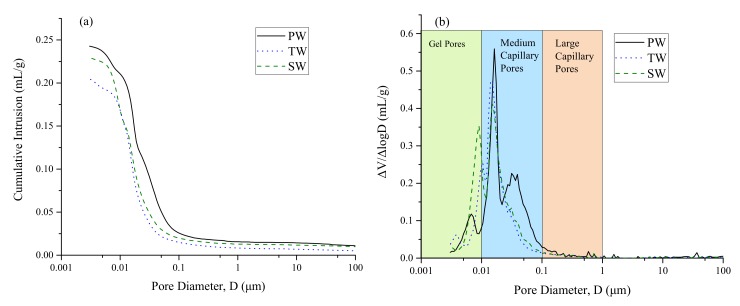
Mercury intrusion porosimetry (MIP) results of AAM produced with different types of water: (**a**) cumulative intrusion of mercury and (**b**) pore size distribution.

Pore size distributions of AAM measured at 28 days are shown in Figure 3. Table 4 presents the total pore area, average pore diameter, and porosity of AAM samples.

The results obtained from MIP show that sea water is beneficial in refining the average pore diameter and porosity of fly ash based AAM. However, an increase of 11.77 m^2^/g in total pore area was observed. It should be noted that in previous studies, no relation between total pore area and strength development was observed [15,16]. The primary cause of such inconsistencies can be induced by the ink bottle effect of MIP method. Furthermore, it is inferred that the complex microstructures of AAM can cause a much higher ink bottle effect. Another aspect of increased porosity in SW samples can be due to the increased amount of gel pores (see Figure 3b). However, it should be noted that these gel pores have almost no role in strength characteristics [17,18]. Nevertheless, the average pore diameter and porosity of AAM samples were reduced by incorporating TW and SW. In addition, it can be observed from Figure 3b that PW-based AAM samples have a larger critical pore diameter along with increased medium capillary pores. Previous studies have reported that higher amount of medium capillary pores resulted in the decreased compressive strength [17,19].

It should be noted that the higher amount of total dissolved solids in SW can play a significant role in lowering the average pore diameter and porosity of AAM samples. Previous studies focused on the use of SW in producing normal Portland based cement systems argued about the densification of cement matrix due to the formation of Friedel’s salt supported by excess amount of sulfate and chloride ions [6,20,21]. It can be stated from the present study that the higher pH in SW could positively influence the development of sodium aluminate silicate hydration (N–A–S–H) which results in the densification of microstructure.

### 3.3. X-Ray Diffraction

**Figure 4:**
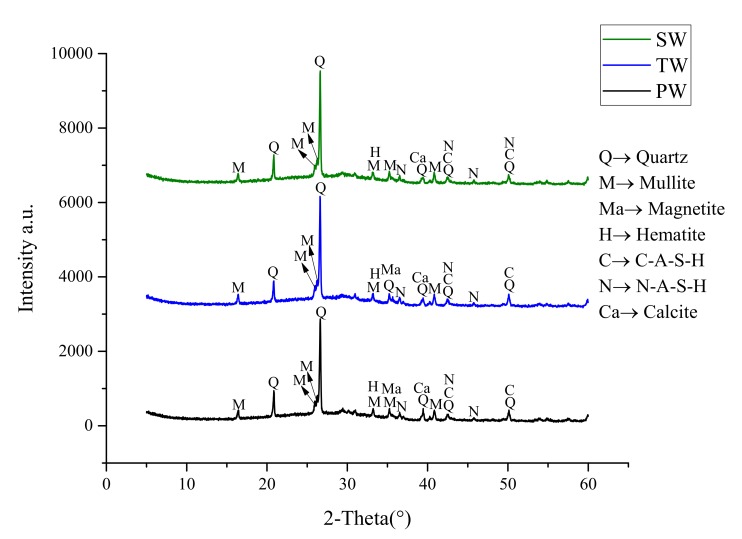
X-ray diffractometry (XRD) spectra of AAM produced with different types of water.

The XRD spectra of AAM samples are presented in Figure 4.

It can be seen that the XRD spectra of AAM made with different types of mixing water are nearly the same with no major differences. The majority of quartz, mullite, magnetite, and hematite observed are from the unreacted crystalline portion of raw fly ash. These phases usually go unaltered due to unreactive nature towards alkali activation. Besides, the hump visible in all XRD spectra at 29°–30° depicts the presence of amorphous unreacted phases of fly ash. As illustrated, two major polymerization phases namely calcium aluminate silicate hydrate (C–A–S–H) and sodium aluminate silicate hydrates (N–A–S–H) were observed in all the samples. These polymeric networks are resulted from dissolution, coagulation and restructuring of the glass phase in fly ash. The formation of C–A–S–H and N–A–S–H are responsible for strength gaining mechanism and can also provide suitable sites for the immobilization of chloride ions [22]. It can be seen that the addition of sea water did not hinder the formation of C–A–S–H and N–A–S–H phases.

The notable difference is the presence of chloride in SW sample that can provide excess chloride ions in the alkali activation process. In a previous study by He et al. [23], the addition of NaCl in slag-based geopolymer resulted in an increased mechanical strength due to the binding of chloride ion to form calcium aluminum chloride sulfate hydrate. In the present study, however, no such phases of chloride-based polymerization products were observed in the XRD spectra. On the other hand, it can be stated that the presence of chloride ions might help in the formation of CaCl_2_ in the matrix. As discussed in Section 3.1, the formation of CaCl_2_ is beneficial in accelerating the early polymerization reaction of AAM which could result in the additional formation of N–A–S–H (as evident in SW spectra from Figure 4). Nevertheless, the important observation can be drawn that the presence of chloride and sulphate ions in SW do not have any negative influence on the mineralogical composition of AAM. 

### 3.4. FT-IR

**Figure 5:**
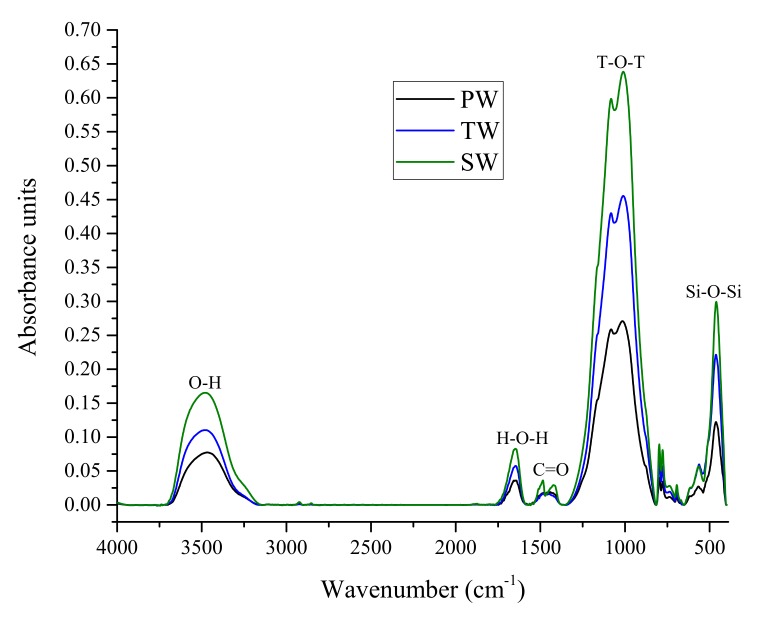
FT-IR spectra of AAM produced with different types of water.

The FT-IR spectra of 28 days AAM samples are presented in Figure 5.

The band observed at 482 cm^−1^ are attributed to the Si–O–Si bond. The Si–O–Si bond band indicates the amorphous nature of fly ash as it’s intensity is independent of crystallization [24]. The split bond band at 1015 cm^−1^ can be identified as T–O–T (T represents Si or Al) bond. The N–A–S–H in alkali-activated fly ash is attributed to the stretching action of T–O–T [24,25]. The bond band at 1482 cm^−1^ is identified as traces of the C=O bond and is a representative of carbonate groups [26]. The stretching and bending vibrations of O–H and H–O–H are represented as weak bands at 1641 cm^−1^ and 3467 cm^−1^. Adsorbed and crystallographically not restrained H_2_O molecules are characterized by O–H and H–O–H, respectively [26].

The primary band of T–O–T is indicative of the polymerization products of alkali-activated fly ash. In all samples, a new low intensity band is observed at 1082 cm^−1^ that specifies the formation of SiQ*_n_* (*n* = 3 or 4). Two reaction mechanisms are primarily responsible for these bands. The more reactive glass phases result in the formation of less polymerized structure at lower wavenumber (1010–1015 cm^−1^), whereas the undissolved quartz phase also undergoes structural changes due to micro-stress [26] leading to a polymerized structure represented by a minor intensity band at 1082 cm^−1^. This splitting of bond bands is in good agreement with previous studies [26,27]. Moreover, the increase in intensity of the T–O–T peak on SW-based sample suggests the increase in the amount (per unit volume) of the functional group associated with the molecular bond. This can lead to improved mechanical property for SW-based AAM.

It can be observed that the addition of sea water did not result in the formation of new bond band, but had minor effect on bond bands. The narrowing of bond bands on the SW-based alkali activation depicts the formation of more condensed reaction products. Furthermore, the slight shift in SW samples to lower wave number of 1010 cm^−1^ (as compared to PW) can be accredited to the formation of N–A–S–H with a higher crosslinking. The presence of the C=O bond for AAM indicates the carbonation reaction between polymerization products and atmospheric carbon dioxide. 

### 3.5. NMR

**Figure 6:**
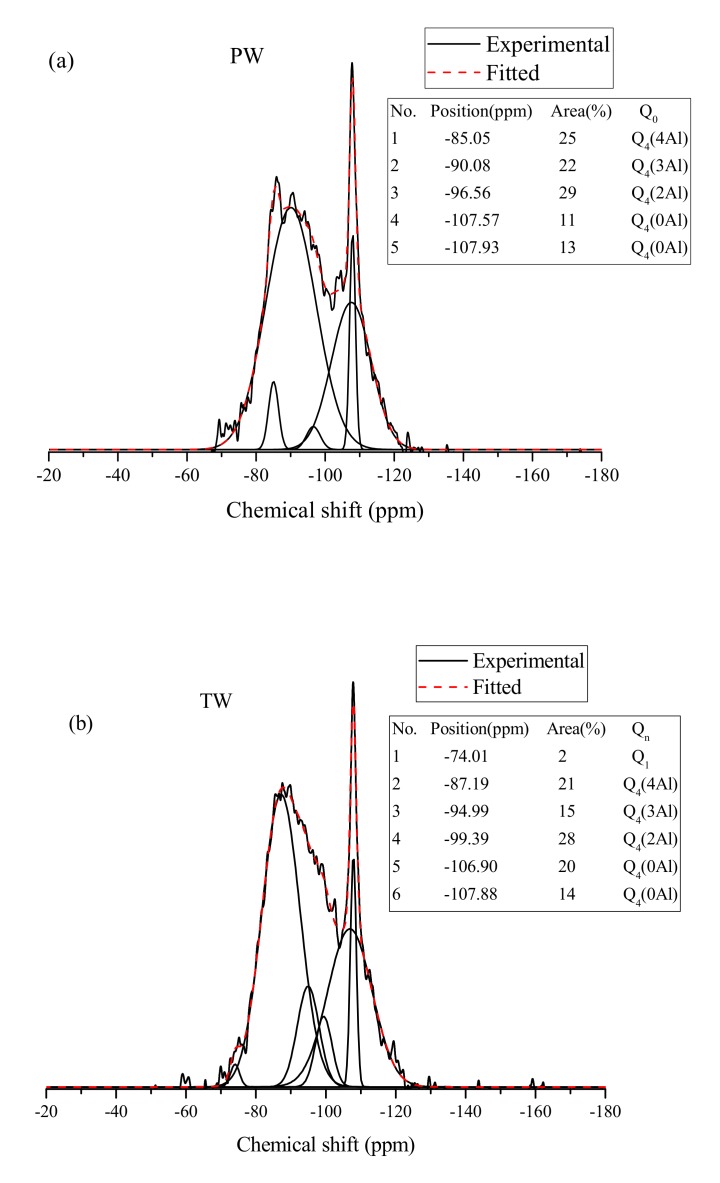

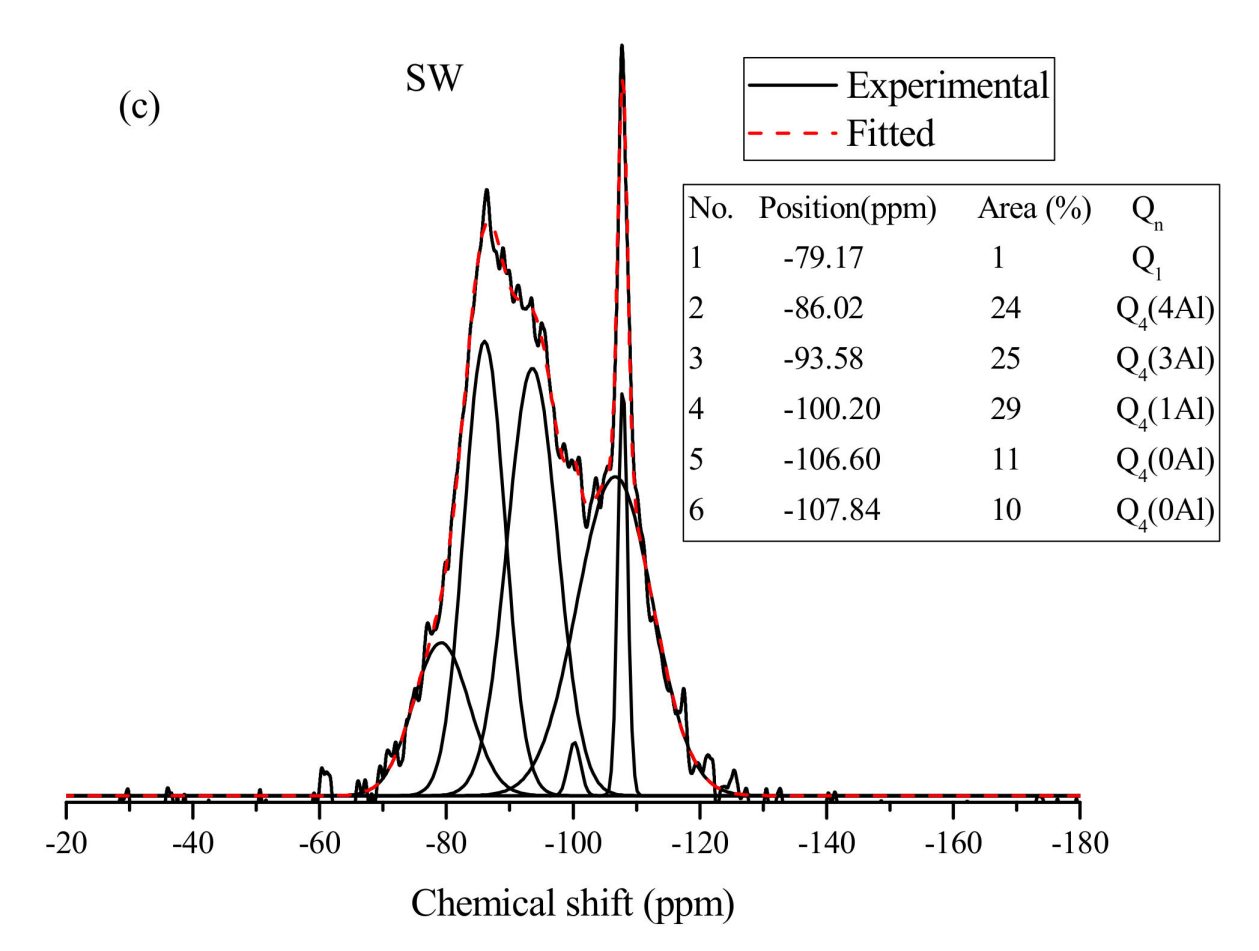
^29^Si NMR spectra and deconvolution results of AAM produced with different types of water: (**a**) PW mix, (**b**) TW mix, and (**c**) SW mix.

Figure 6 depicts the ^29^Si NMR spectra of AAM prepared with PW, TW, and SW. In the present study, the Origin software package was used to deconvolute the NMR spectra.

The notation Q*_n_*(*m*Al) is used to depict the chemical bonds of the resonating Si nuclei where ‘*n*’ represents the number of adjacent tetrahedral SiO_4_ linked to a specific SiO_4_ tetrahedron, and *m* denotes the number of Al substitution to the corresponding Si tetrahedra [28,29,30,31,32,33,34]. The ratio of Si/Al (only Q_4_(*m*Al)) for different AAM is provided in Table 5, and the ratio was calculated by referring to previous studies [32,35].

As seen in Figure 6, the AAM produced from various water sources has quite similar spectra signifying that the use of TW and SW did not significantly affect the polymerization process. Moreover, the use of SW leads to the higher Si/Al ratio as compared to TW and PW mixes as shown in Table 5. This confirms the findings of a previous study by Kovalchuk et al. [25] that the Si/Al ratio is directly proportional to a mechanical strength. It should be noted that presence of alkali cation such as Na^+^ influences the alkali activation process of AAM. In the present study, the SW has a higher amount of Na^+^ than PW and TW. This promotes the dissolution of Si and Al which results in higher Si/Al in the SW based AAM. Similar observations were drawn by Peng et al. on investigating the influence of alkali cation on microstructure of AAM [36]. Additionally, from Figure 6, it can be observed that the intensity of peaks after deconvolution depicts a slightly better structured spectra for SW-based AAM as compared to PW- and TW-based mixes. The broad and poorly defined peaks in PW and TW reflect a disorder gel structure affecting the mechanical performance. However, the presence of Q_1_ peak intensity for TW and SW mixes indicates the presence of hydrolyzed material that is yet to undergo polymerization into Q_4_(*m*Al) structures. This can be attributed by the difference of water molecules present in TW and SW due to dissolved salts. These dissolved salts are responsible for disbalance of Si/Na ratio, as excess content usually leads to faster condensation which can slightly delay further polymerization. In summary, the use of SW does not have a detrimental effect on the polymerization process of AAM mixes.

### 3.6. Free Chloride Content

Free chloride contents in the AAM mixes are presented in Table 6.

Although the SW-based AAM sample had the highest content of free chlorides, a high chloride binding capacity of AAM is observed. The chloride binding capacity of AAM is governed by the encapsulation process within the matrix [37]. In the case of the SW-based AAM, nearly 86.8% of chloride ion was bound by alkali activation in this study. The exact binding mechanism of chloride is still not clear due to its complex nature. However, it is interesting to note that the increase of free chloride contents in PW and TW samples were observed. It can be theorized that the unconsumed Na^+^ from the alkali-activating solution can form NaCl by reacting with chloride ions, and then deposited on the N–A–S–H gel surface as precipitation. These NaCl precipitates formed on surface can be measured during the free chloride measurements. The chloride present in SW-based AAM influences the polymerization process by forming CaCl_2_ and NaCl precipitates. The pore solution of N–A–S–H gels then absorbs or encapsulates these chloride precipitates within the binder matrix, thereby immobilizing them which can influence the free chloride content. A similar observation was also drawn by Shi et al. on incorporating sea water to produce alkali activated calcium silicate slag [9]. In addition, the authors would like to point out that as compared to PW and TW, the SW also has a high amount of sodium (Table 3), which can enhance the amount of unconsumed Na^+^ in the alkali-activating solution.

The mechanism of chloride binding needs to be further investigated. Previous studies also reported that the absence of Friedel’s salt in AAM can be due to preferred formation of zeolitic phases and N–A–S–H gel [22]. The absence of AFm phase is responsible for the non-existence of Friedel’s salt. It should also be noted that the present study focused on the binding behavior of chloride ions already present in the alkali-activating solution rather than the ingress of chloride ions in hardened AAM paste. The finding of the free chloride content in SW-based AAM suggests that caution is necessary before incorporation in steel reinforced structural elements.

## 4. Conclusions

The present study investigated the compressive strength, microstructure, mineralogy, and free chloride content of alkali-activated fly ash material produced from pure water, tap water, and sea water. The main findings of the study are summarized below.

1. The difference in compressive strength was marginal on utilizing the three different types of water for alkali activating solution. Moreover, the presence of chloride ions and higher pH of sea water were instrumental for slightly greater gain in compressive strength.
2. The use of sea water resulted in the refined pore structure along with reduced average pore diameter. The primary cause was the higher amount of polymerization products that densified the matrix.
3. The XRD results showed that the use of sea water has negligible effects on the mineralogical phases of alkali-activated fly ash material. Moreover, the absence of any chloride and sulphate based crystalline minerals is an evidence of the immobilization potential of the alkali activation process.
4. The FT-IR spectra of the alkali-activated samples showed no negative influence of sea water on the bond band of polymerization products. The results suggest that the use of sea water leads to higher crosslinking of sodium aluminosilicates hydrates in alkali-activated fly ash material.
5. The ordering structure and higher Si/Al ratio observed from 29Si NMR spectra showed that the sea water-based alkali-activated fly ash material has higher content of Q4 groups. Furthermore, the sea water-based alkali-activated fly ash material has the higher formation of zeolitic Si–O–Al linkages which is indicative of more matured paste matrix.

## Figures and Tables

**Table 1:** Chemical composition of fly ash.

Oxide Composition	Fly Ash (wt.%)
SiO_2_	52.50
CaO	5.04
Al_2_O_3_	24.80
Fe_2_O_3_	6.00
SO_3_	1.00
MgO	1.56
P_2_O_5_	0.70
K_2_O	1.50
Na_2_O	0.90
TiO_2_	1.40
SrO	0.20
BaO	0.20
Loss of ignition	4.20

**Table 2:** Composition (ppm) and pH of pure water, tap water, and sea water.

Compound	Pure Water	Tap Water	Sea Water
F	0.05	0.18	4.03
Cl	0.18	52.28	41,942.03
NO_2_	-	-	-
Br	-	0.15	110.86
NO_3_	0.29	12.66	158.46
PO_4_	-	-	-
SO_4_	0.75	39.35	6802.00
pH	7.00	7.71	8.38

**Table 3:** Cations concentration (ppm) of pure water, tap water, and sea water.

Element	Pure Water	Tap Water	Sea Water
Al	<0.010	<0.010	0.106
Ba	<0.005	<0.005	<0.005
Ca	1.238	23.07	381.4
K	<0.010	1.981	316.2
Mg	<0.005	4.516	1129
Na	0.407	8.419	7359

**Table 4:** Total pore area, average pore diameter and porosity of AAM produced with different types of water.

Sample	Total Pore Area (m^2^/g)	Average Pore Diameter (nm)	Porosity (%)
PW	54.80	17.70	35.84
TW	59.25	13.80	32.53
SW	66.57	13.80	34.95

**Table 5:** Si/Al ratio of Si-O-Al linkage in different AAM mixes.

Sample	Si/Al Ratio
PW	1.96
TW	1.91
SW	2.08

**Table 6:** Free chloride content in AAM mixes obtained by ion chromatography.

Sample	PW	TW	SW
Free chloride content (ppm)	407.22	528.64	5535.00
